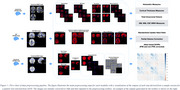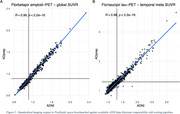# ADprep – A Fully‐Automated Software for Large‐scale Multimodal MRI and PET Imaging Workflows

**DOI:** 10.1002/alz70856_101373

**Published:** 2025-12-25

**Authors:** Amir Dehsarvi, Lukas Frontzkowski, Anna Dewenter, Michael Schöll, Nicolai Franzmeier

**Affiliations:** ^1^ Institute for Stroke and Dementia Research (ISD), University Hospital, LMU Munich, Munich, Bavaria, Germany; ^2^ Department of Psychiatry and Neurochemistry, Institute of Neuroscience and Physiology, The Sahlgrenska Academy, University of Gothenburg, Mölndal, Sweden; ^3^ Wallenberg Centre for Molecular and Translational Medicine, Gothenburg, Sweden; ^4^ Dementia Research Centre, UCL Queen Square Institute of Neurology, London, United Kingdom; ^5^ Institute for Stroke and Dementia Research (ISD), LMU University Hospital, LMU, Munich, Bavaria, Germany; ^6^ University of Gothenburg, The Sahlgrenska Academy, Institute of Neuroscience and Physiology, Psychiatry and Neurochemistry, Gothenburg, Sweden; ^7^ Munich Cluster for Systems Neurology (SyNergy), Munich, Bavaria, Germany

## Abstract

**Background:**

Processing of large‐scale multimodal MRI and PET data is crucial for advancing Alzheimer's disease (AD) neuroimaging research. However, image processing requires strong expertise on different software packages (SPM/FSL/AFNI/FreeSurfer) and programming languages (R/MATLAB/Python/Bash) and lab‐specific image processing approaches are a major roadblock for harmonization across sites. Therefore, establishing uniform and user‐friendly image processing workflows is crucial for inter‐site standardization and harmonization of neuroimaging data and to reduce bias introduced by different preprocessing strategies. Therefore, we developed the containerized, state‐of‐the‐art, fully automated, neuroimaging toolbox ADprep that integrates robust preprocessing of structural/functional MRI, and multi‐tracer PET (amyloid/tau/FDG/TSPO), generating standardized nifti and atlas‐based spreadsheet outputs across a broad range of brain atlases. ADprep requires no programming expertise and can facilitate harmonized neuroimaging analyses and data sharing across the AD neuroimaging community and will be fully integrated into the cloud‐based GRIP platform.

**Methods:**

ADprep works on bids‐formatted data and was fully developed in nipype (Figure 1). Preprocessing for structural MRI includes volumetric and cortical thickness assessments for widely used brain atlases (Desikan‐Killiany/Schaefer100‐600/LPBA/Hammers/Neuromorphometrics/Cobra/Destrieux), plus spatially normalized and smoothed tissue segments for voxel‐based morphometry analyses. Functional MRI processing includes slice‐timing and motion correction, nuisance regression, spatial normalization, and functional connectivity assessments for above‐ mentioned atlases. PET processing includes generation of spatially normalized SUVR images for different tracer‐specific reference regions, as well as extraction of atlas‐based SUVRs and partial‐volume correction. Standardized outputs in FreeSurfer space were benchmarked against data from the ADNI imaging core to illustrate comparability with existing pipelines. Cloud‐based GRIP and local cluster implementation is provided to ensure large‐scale data processing.

**Results:**

ADprep was tested successfully on large‐scale multimodal datasets, including several thousand scans from ADNI, ADNI‐DOD, and A4 with an overall processing failure rate of <4%. Using data from the ADNI PET core for benchmarking, ADprep closely reproduces openly available amyloid‐PET (r=0.99, *p* <0.001, Figure 2A) and tau‐PET SUVRs (r=0.98, *p* <0.001, Figure 2B). Runtime is ∼1h for a structural/functional MRI and ∼30min for a PET scan.

**Conclusions:**

ADprep is user‐friendly and harmonized multimodal neuroimaging pipeline, that can be applied to different neuroimaging datasets by non‐expert users, providing outputs that can be directly used for statistical analyses.